# Investigation of the correlation between mood disorder symptoms and disease activity and functional status in rheumatoid arthritis patients

**DOI:** 10.3906/sag-2107-283

**Published:** 2021-11-04

**Authors:** Melih PAMUKCU, Tuğba İZCİ DURAN, Hasan ULUSOY, Kürşat ALTINBAŞ

**Affiliations:** 1 Division of Rheumatology, Department of Internal Medicine, Dışkapı Yıldırım Beyazıt Education and Research Hospital, Health Sciences University, Ankara, Turkey; 2 Division of Rheumatology, Department of Internal Medicine, Faculty of Medicine, Ondokuz Mayıs University, Samsun, Turkey; 3 Division of Rheumatology, Department of Internal Medicine, Faculty of Medicine, Ondokuz Mayıs University, Samsun, Turkey; 4 Department of Psychiatry, Faculty of Medicine, Selçuk University, Konya, Turkey

**Keywords:** Arthritis, rheumatoid, bipolar disorder/psychology, depression/psychology, prevalence

## Abstract

**Background/aim:**

To investigate the correlation between depressive-anxiety symptoms, mixed features, disease activity, and functional status in patients with rheumatoid arthritis (RA) in the light of the shared underlying etiology in both disorders.

**Materials and methods:**

The study included 556 patients with RA. RA disease activity was measured using the Disease Activity Score 28-joint count C reactive protein (DAS28-CRP), and the patients were evaluated by a Health Assessment Questionnaire (HAQ). The Hospital Anxiety and Depression Scale (HADS), Mood Disorder Questionnaire (MDQ), and Modified Hypomania Checklist (mHCL) were used to evaluate the mixed depression and bipolarity status of the patients.

**Results:**

Of the patients, 430 (77.3%) were female and 126 (22.7%) were male. The median age was 57 years, the median HAQ score was 0.55 points, and the median DAS28-CRP score was 4.1 points. The evaluation of the patients by DAS28-CRP revealed that 58.5% of the patients had moderate and severe disease activity, while only 23.4% of them were in remission. The group using the combination of synthetic disease-modifying anti-rheumatic drugs (sDMARD) and steroid therapy had significantly higher HAD-depression, HAD-anxiety, mHCL, DAS28-CRP, HAQ, and MDQ scores than the group using sDMARD alone. The grouping of the patients based on the DAS28-CRP cut-off scores showed that the patients with moderate and severe disease activity had significantly higher HADS, mHCL, MDQ scores than those in remission and those with mild disease activity (p < 0.001).

**Conclusion:**

Disease severity and functional status in RA can be affected by comorbid anxiety-depressive and mixed symptoms. Therefore, clinicians should consider screening the depressive-anxiety and mixed mood symptoms of RA patients. Moreover, patients who use steroid therapy are more susceptible to mood symptoms (anxiety, depression, bipolarity), which should also be considered during the follow-up of patients.

## 1. Introduction

Rheumatoid arthritis (RA) is a chronic inflammatory autoimmune disease characterized by inflammation in the synovium of the joints and destruction of the cartilage and bone in the affected joints [1]. The primary presentation of the disease is joint involvement and is essential for the diagnosis of the disease. Nevertheless, there are many other associated systemic symptoms and comorbidities affecting the functional status and quality of life of RA patients [2]. RA patients commonly have comorbid psychiatric illnesses such as anxiety and depressive disorders. However, the etiology underlying this comorbidity has not been fully understood [3].

Chronic inflammation has a major role in the pathophysiology of depression, especially for mood disorders [4]. Many studies investigating the pathogenesis of RA symptoms have reported chronic inflammation and increased cytokine levels in RA patients compared to healthy controls [5]. Autoantibodies found in the serum of patients with RA have provided many clues for the pathophysiology [6]. RA patients usually have infiltration of joint tissue (synovium) by immune cells such as B cells, T cells, and macrophages that release a range of proinflammatory cytokines and promote inflammation, resulting in tissue destruction. Although epidemiological studies have identified environmental and genetic factors contributing to the risk of developing RA, the exact cause of autoimmune response in these patients has not yet been fully elucidated [7]. On the other hand, studies have shown a correlation between autoimmune and psychiatric diseases, especially bipolar disorder, major depressive disorder, and schizophrenia [8].

Long-term treatment plays a crucial role in chronic diseases and positive clinical outcomes of patients suffering from these conditions including mood disorders and RA depend on their adherence to treatment. Although the prognosis of RA and treatment adherence are affected by comorbid psychiatric illnesses [9], elimination of psychiatric conditions has been shown to increase the treatment success for RA [10]. There are numerous studies in the literature investigating depressive symptoms in RA. However, to the best of our knowledge, there is no study on mixed depression in RA. Depression with mixed features has been included in the Diagnostic and Statistical Manual of Mental Disorders, Fifth Edition (DSM-5) [11]. Recent studies have shown a more severe course of mixed depression and poorer treatment response compared to pure depression [12]. Therefore, this study aimed to investigate the correlation between depressive-anxiety symptoms, mixed features, disease activity, and functional status in patients with RA in the light of the shared underlying etiology in both disorders.

## 2. Materials and methods

### 2.1. Study design

This cross-sectional study included 556 patients, who presented to the rheumatology department between 1 September and 31 December 2020 and who were diagnosed with RA according to the 2010 College of Rheumatology / European League Against Rheumatism (ACR/EULAR) classification criteria [13].

### 2.2. Participants

The study inclusion criteria were set as follows: patients aged 18 years and over who were regularly followed up and treated for RA in the rheumatology clinic, who did not have a history of alcohol and substance abuse, and who were not on psychiatric medication.

The exclusion criteria were set as follows: patients aged under 18 years, who were on psychiatric therapy, had a history of alcohol and substance abuse, other uncontrolled medical disorders, mental retardation and overlap syndromes with RA. Patients were included in the study consecutively according to inclusion and exclusion criteria.

### 2.3. Data collection

All patients’ demographic characteristics and clinical data were analyzed. The clinical data included duration of disease, drugs used at the time of admission and before, habits (smoking, alcohol, etc.), and history of other systemic diseases. Laboratory analysis was performed to determine C-reactive protein (CRP), albumin (g/dL) levels, and erythrocyte sedimentation rate (ESR). Complete blood count analysis was performed to leukocyte, neutrophil, lymphocyte, and thrombocyte count.

All patients underwent systemic and rheumatologic physical examinations. RA disease activity was measured using the Disease Activity Score 28-joint count-CRP (DAS28-CRP). Moreover, the patients were evaluated by a Health Assessment Questionnaire (HAQ). DAS28-CRP and HAQ scores were calculated by a rheumatologist. The mixed depression and bipolarity states of the patients were evaluated using individual and anonymous questionnaires. All measurement tools used in the study have been evaluated by the Turkish validity and reliability studies.

### 2.4. Measurement tools

#### 2.4.1. Hospital anxiety and depression scale (HADS)

HADS is a self-report questionnaire developed by Zigmond and Snaith in 1983 to measure levels of anxiety and depression [14]. It consists of 14 items. Seven of the items (odd numbers) measure anxiety, while the other seven items (even numbers) measure depression. It is a useful scale to screen depression and anxiety states of patients with chronic diseases who are treated in outpatient and clinical settings. Its design offers ease of use for nonpsychiatric departments. However, a complete clinical evaluation is required to make the definitive diagnosis of anxiety and depression. Each item of this scale is rated on a 4-point Likert scale (0–3), with scores ranging between 0 and 21 points for each of the two subscales. A score between 0 and 7 indicates a normal state, while a score of 8 and above indicates anxiety and depression symptoms. Aydemir et al. conducted the Turkish validity and reliability study on the questionnaire [15].

#### 2.4.2. Modified Hypomania Checklist (mHCL)

mHCL is a self-rating screening tool for evaluating manic symptoms in current depressive episode [16]. mHCL consists of 32 questions to screen manic and hypomanic symptoms. There is no cut-off score for m-HCL-32. At least three manic symptoms are required for mixed depression diagnosis according to the DSM-5 [11]. Altinbaş et al. conducted the Turkish validity and reliability study on the checklist [16].

#### 2.4.3. Mood Disorder Questionnaire (MDQ)

MDQ is a self-assessment instrument developed by Hirschfeld et al. in 2003 [17]. It consists of 17 questions to screen bipolar disorder. In the questionnaire, 13 questions about possible symptoms are answered either “yes” or “no”, and 4 questions assess the level of functioning, cooccurring symptoms, previous diagnosis of BD, and family history of bipolar disorder (BP). The criteria of at least 7 or more symptoms, two or more cooccurring symptoms, and the presence of moderate or severe impairment should be met in order to evaluate screening positive (MDQ standard cut-off). The cut-off value for the questionnaire has been reported as 7, with a sensitivity of 0.64 and a specificity of 0.77. Konuk et al. conducted the Turkish validity and reliability study on the questionnaire [18].

#### 2.4.4. Disease Activity Score 28-joint count C reactive protein (DAS28-CRP)

DAS28-CRP is used to determine the severity of RA using CRP along with the number of sensitive and swollen joints. The number of swollen joints is determined by a visual analog scale and CRP levels. The DAS28-CRP score ranges between 0 and 9.4, with <2.6 indicating remission, ≥2.6 and ≤3.2 low disease activity, >3.2 and ≤5.1 moderate disease activity, and >5.1 indicating severe disease activity.

#### 2.4.5. Health Assessment Questionnaire (HAQ)

HAQ is a comprehensive instrument designed to evaluate a patient’s health status. HAQ is one of the measures of the ACR Core Data Set for the assessment of RA disease activity and patient-oriented outcomes, including disability, drug-associated side effects, discomfort, cost of care, and mortality. It includes 20 items divided into the eight subcategories of dressing, arising, eating, walking, hygiene, reaching, gripping, and usual activities to determine patients’ ability to use upper or lower limbs. Each item of HAQ is rated on a 4-point scale ranging from 0 to 3, where 0 = without any difficulty, 1=with some difficulty, 2=with much difficulty, and 3=unable to do. The final HAQ index ranges from 0 to 3 and is scored by averaging the items from all eight categories. A HAQ score <0.3 is considered normal; however, the average HAQ of the population has been shown to increase with increasing age [19]. A higher HAQ score indicates higher disability. In RA patients, the minimum clinically significant difference in serial measurement of HAQ scores has been shown to be 0.22 at the group level [19].

### 2.5. Statistical analysis

SPSS V22.0 statistical software package (SPSS, Inc., Chicago, IL, USA) was used for all data analyses. Categorical variables were presented as numbers (percentage). Numerical variables were presented as mean ± standard deviation (SD) or median (min – max) according to their distribution. The Kolmogorov–Smirnov test was used to check the normality assumption of numeric variables. The independent-samples t-test was used for intergroup comparisons of normally distributed numeric variables, while the Mann–Whitney’s U test was used for nonnormally distributed variables. The chi-square test was used to evaluate categorical data. Three groups’ comparisons were done with Kruskall–Wallis test. Posthoc analyses were performed by Mann–Whitney U test with Bonferroni correction, and the p value p < 0.05 / 3 = 0.017 was considered as significant. The odds ratio (OR) with a 95% confidence interval (CI) was used to report the strength of association. Spearman’s correlation analysis (r) was used for correlation analysis. Factors affecting anxiety, depression, mixed depression, and bipolarity were analyzed using the backward linear regression analysis. Variables found to be statistically significant in univariate analyses were included in linear regression analysis. Correlations between risk factors and outcomes are presented as odds ratios (ORs) and 95% CI. The level of statistical significance was set at p < 0.05.

## 3. Results

### 3.1. Sociodemographic and clinical characteristics of patients

Distribution of sex in our samples revealed that 430 (77.3%) were women. The median age of the patients was 57 years, and the median age at diagnosis was 57 years. The median disease duration was 4 years. The assessment of the patients’ disease activity and functional status revealed a median HAQ score of 0.55 and a median DAS28-CRP score of 4.1 points. Of the patients, 386 (69.4%) had positive RF and 371 (66.7%) had positive anti-CCP. The treatment regimens of the patients were also evaluated. Three hundred and eleven of the patients were on steroids, with a median steroid dose of 2.5 and a median duration of steroid use of 2 years. Of the patients, 207 (37.2%) were using synthetic disease-modifying anti-rheumatic drugs (sDMARD), while 37 (6.7%) were using sDMARD with biologic DMARD (bDMARD). One patient (0.2%) was receiving only bDMARD. The clinical and laboratory characteristics of the patients are summarized in Table 1. A total of 24 patients were excluded from the study due to psychiatric treatment.

### 3.2. Comparison of Mood Disorder Questionnaire Scores by treatment regimens and disease activity

Of the patients, 253 (45.5%) had depression, 216 (38.8%) had anxiety, and 194 (34.9%) had mixed depression according to the cut-off scores of psychiatric screening scales. The comparison of the patients’ psychiatric scale scores by treatments are shown in Table 2.

The comparison of the HAD-depression (HAD-D), HAD-anxiety (HAD-A), mHCL, DAS28-CRP, HAQ, and mood disorders scale scores of the patients who were on sDMARD or on sDMARD in combination with steroid therapy revealed that the patients receiving sDMARD plus steroid therapy had significantly higher scores in all scales.

The sample was divided into three groups based on their DAS28-CRP scores. The patients with moderate and severe disease activity had significantly higher HADS, mHCL, MDQ scores than both those in remission and those with mild disease activity (p < 0.001). However, there was no significant difference between the remission group and the mild disease activity group in terms of psychiatric scale scores. (Table 3).

### 3.3. Correlation and regression analysis of rheumatological and psychiatric scale scores

The evaluation of the correlations between mood disorders and RA disease activity revealed a moderate correlation between MDQ and HAQ scores (r = 0.41, p < 0.001) and between MDQ and DAS28-CRP scores (r = 0.59, p < 0.001). Moreover, there was a moderate correlation between HAD-D and HAQ scores (r = 0.39, p < 0.001) and between HAD-D and DAS28-CRPscores (r = 0.60, p < 0.001). Furthermore, the evaluation of the correlations of HAD-A showed a moderate correlation with the HAQ score (r = 0.36, p < 0.001) and a moderate correlation with DAS28-CRP (r = 0.50, p < 0.001). The evaluation of the correlations of mHCL revealed a moderate correlation with HAQ score (r = 0.41, p < 0.001) and a moderate correlation with DAS28-CRP (r = 0.59, p < 0.001).

The linear regression analysis of the patient groups by HAQ and DAS28-CRP scores showed that DAS28-CRP scores increased with the increase in mHCL, HAD-D, and MDQ scores and that an increase in HAQ scores was associated with the increase in mHCL scores (Table 4).

## 4. Discussion

The present study demonstrated that the group using sDMARD plus steroid therapy had significantly higher all mood disorder scale scores such as HADS-D, HADS-A, mHCL, MDQ, and disease activity and functional status scale scores such as DAS28-CRP and HAQ compared to the group receiving sDMARD monotherapy. The grouping of the patients based on the DAS28-CRP cut-off scores showed that the patients with moderate and severe disease activity had significantly higher HADS, mHCL, MDQ scores than those in remission and those with mild disease activity. The higher depression and bipolarity scores of the patients receiving steroid therapy indicate an association between steroid therapy and psychiatric symptoms.

The prevalence of psychiatric symptoms is high in inflammatory chronic conditions such as RA. A metaanalysis of 72 studies, including a total of 13189 patients, reported a depression prevalence between 9.5% [20] and 41.5% [21] in patients with RA. In their systematic review and metaanalysis of a small number of studies using gold-standard clinical interviews, Matcham et al. found that 16.8% of RA patients, mostly consisting of female and older adult patients, had major depression [22]. A Taiwan study by Hsu et al. comparing 2570 RA patients with 2570 controls without RA found a higher incidence of bipolar disorder (OR = 2.13, 95% CI=1.12–4.24, p=0.013) in RA patients compared to controls [23]. Another study comparing 11782 RA patients with 57973 age- and sex-matched controls found a higher prevalence of bipolar disorder in RA patients compared to controls [24]. A metaanalysis by Charoenngam N. et al. reported an approximately two-fold higher frequency of bipolar disorder development in patients with RA compared to the healthy population [25]. We found that nearly half of our sample reported higher depression scores, while one-third has probable anxiety disorders according to the HADS cut-off scores. Besides, nearly one-third of the patients with RA reported higher scores on MDQ cut-off score. These relatively high scores of mood disorder scales might be due to the self-rating nature of the scales. Such varying results between studies may be due to the diagnostic criteria of depression used by different studies as well as the high number of psychometric instruments to screen depression [26]. Nevertheless, the bidirectional relationship between mood disorders and inflammatory autoimmune diseases such as RA should be further studied in long-term follow-up studies.

Inflammation is the underlying mechanism proposed for both RA and mood disorders [8,9]. RA is a well-defined, immune-mediated chronic inflammatory disease characterized by increased levels of proinflammatory cytokines, including interleukin 6 (IL6), interleukin 1ß (IL1ß), and tumor necrosis factor 1α (TNFα) [27]. Although the pathogenesis of depression has not been fully elucidated, the effect of immune-mediated pathways on the pathogenesis has been the subject of many studies [28]. Metaanalyses have shown higher concentrations of cytokines such as IL1ß, TNFα, and IL6 in the peripheral blood of patients with depression compared to healthy controls [28–30]. Symptoms associated with depressive disorders such as asthenia and depressed mood pose a major health burden for many countries, particularly for those with chronic diseases such as spondyloarthritis and RA [31]. Patients suffering from joint diseases usually manifest these symptoms, affecting their productivity and quality of life [32]. Depression is the most common psychiatric disorder accompanying RA due to a probable increase in the prevalence of fatigue, pain, poor health-related quality of life, physical disabilities, and increased cost of care [30]. In addition to the impact of RA causing depressive symptoms, chronic inflammation has been shown to have a major contribution to the pathophysiology of mood disorders such as bipolar and depressive disorder [4,33]. Although the exact mechanism of this condition is unknown, there are various hypotheses. One of these hypotheses is chronic neuroinflammation involved in the pathogenesis of bipolar disorder [34]. Numerous studies have shown increased levels of peripheral inflammatory markers such as TNFα, IL4, IL6, and IL10 in bipolar patients [35]. Poor quality of life and functional impairment have been reported as risk factors for the development of anxiety disorder and major depressive disorder in RA patients [36]. Our study showed that patients with RA, who were receiving steroid therapy and had moderate and severe disease activity, had higher psychiatric scale scores, which is consistent with the previously suggested hypotheses about the higher prevalence of bipolar disorder in RA. However, the multivariate analysis revealed an insignificant correlation between bipolar disorder and RA [24]. Moreover, the levels of inflammatory cytokine were not evaluated in our study, which can be considered as a limitation.

The importance of identifying and measuring depression in patients with RA is not only to treat a worsening comorbid disease but also to prevent the functional decline and decrease in response to treatment caused by depression in patients with RA [37]. Depression may mitigate with remission of rheumatoid arthritis [38]. Furthermore, depression treatments have previously been shown to support this immuno-psychiatric association, for example, it has been shown that antidepressants lower inflammation, while a high level of inflammation at baseline predicts a lower treatment efficacy for most treatments [39]. In our study, depression scales were found to be lower in patients with remission and lower disease activity. Depression has been shown to increase the limitation of movement [40] and reduce treatment response and remission [41] in RA patients. Furthermore, it has also been reported that depression reduces the continuity of anti-rheumatic drugs [41]. Previous studies have attributed this to different reasons [42]. Other comorbid conditions such as cardiovascular diseases associated with RA are also often associated with depression [43]. Depression has been shown to increase suicidal [44] and nonsuicidal mortality [45] in RA patients. Our results revealed that depression was strongly correlated with the disease activity and severity in RA. However, the cross-sectional design of our study does not allow us to conclude that this result is the cause or consequence. There is a need for further cohort follow-up studies with a larger sample size to find an answer to this question. On the other hand, we did not find a correlation between the HAQ score and the MDQ score. The fact that MDQ is not a highly selective scale in terms of sensitivity and specificity and is used to screen the general population may be the reason for the absence of a significant correlation. However, 32 hypomanic-manic symptoms are screened on the mHCL scale. Increased hypomanic-manic symptoms and higher screening scores are significantly correlated with the HAQ score. Manic symptoms in bipolar disorder may indicate a more severe disease course. Therefore, the fact that depression was not found to be significant might be associated with the abovementioned reasons.

The correlation between steroid exposure and neuropsychiatric disorders has been investigated in many studies and it has been shown that high-dose steroid exposure such as Cushing’s syndrome is associated with behavioral disorders ranging from severe depression to mania [19]. Steroids are frequently used to suppress inflammation in patients with RA, the psychiatric and cognitive effects of which are well defined [46]. Depression, mania, and hypomania, mania, the primary symptoms of bipolar disorder, have also been reported to arise with steroid use [47]. A prospective study reported depression and hypomania symptoms in 10% and 26% of the patients, respectively, who received steroid therapy for 8 days [48]. Although not clearly demonstrated, the potential mechanism of action is the toxic effect of steroids on hippocampal neurons and other brain regions [49], the presynaptic effects on dopaminergic and cholinergic neurons [50], and the inhibition of serotonin release [51]. In line with this evidence, we found that the depression, anxiety, and hypomanic symptom scores were higher in the DMARD plus steroid group and severe disease activity. This result might be the consequence of steroid therapy. Many confounding factors such as psychosocial stressors and inflammatory changes as a shared etiologic factor for both disorders could not be excluded.

Our study has several limitations. First, the cross-sectional nature of the study is the major limitation. Second, RA patients were recruited from a single center, which limits the generalizability of the results despite the relatively big sample size of the study. Third, there was no structured psychiatric interview for the patients, and only screening tools were used to identify psychiatric symptoms. Fourth, the heterogeneity of the sample is another limitation, considering that most of the patients had advanced disease.

In conclusion, both mood disorders and RA are diseases in which inflammatory processes continue their existence as common etiological pathways. Disease severity and functional status in RA can be affected by comorbid anxiety-depressive and mixed symptoms, as demonstrated by this study. Therefore, clinicians should consider screening the depressive-anxiety and mixed mood symptoms of RA patients. Moreover, patients who use steroid therapy are more susceptible to mood symptoms (anxiety, depression, bipolarity), which should also be considered during the follow-up of patients. There is a need for long-term controlled studies with a larger sample size to evaluate the impact of psychiatric comorbidities and the casual relationship with steroid use in RA.

To sum up,

-Depressive, anxiety and mixed mood symptoms are common in RA patients.
-Psychiatric symptoms are associated with disease activity and functionality.
-Patients on steroid treatment are more vulnerable to represent all type of mood symptoms.
-Clinicians should consider the impact of mood symptoms during the diagnosis and treatment process of RA.

## Figures and Tables

**Table 1:** The patients’ features and laboratory values.

Female, n (%)		430 (77.3%)
Age, years		57 (20–81)
Disease Onset Age		52 (17–80)
Disease duration (years)		4 (0.5–25)
Rheumatoid factor, n (%)		386 (69.4%)
Anti–citrullinated protein antibody, n (%)		371 (66.7%)
WBC		7.2 (1.0–21.5)
Neutrophil (10^3^ / ϻL)		4.2 (1.1–55.6)
Lymphocyte (10^3^ / ϻL)		2.0 (0.7–9.7)
CRP		6.3 (0.03–112.1)
ESR		16 (2–67)
Albumin (g / L)		4.3 (2.5–5.1)
HAQ score		0.55 (0.05–2.2)
DAS28-CRP		4.1 (1.2–6.4)
Disease activity according to DAS28-CRP	Remission	130 (23.4%)
	Mild	101 (18.2%)
	Moderate - Severe	325 (58.5%)
Treatment regimens	sDMARD	207 (37.2%)
	sDMARD + Steroid	285 (51.3%)
	bDMARD + sDMARD	37 (6.7%)
	bDMARD + sDMARD + Steroid	14 (2.5%)
	bDMARD	1 (0.2%)
	Streoid	12 (2.2%)

Categorical variables expressed as n (%)

Continuous variables expressed as median (min-max) values

*bDMARD* Biologic Disease Modyfiying Anti-Rheumatic Drugs, *CRP* C-Reactive protein (mg/dL, normal range 0–5), *DAS28-CRP* Disease Activity Score28-C-Reactive protein, *ESR* Erythrocyte Sedimentation Rate (mm/h; normal range 0–20), *HAQ* Health Assessment Questionnaire, *NLR* Neutrophil / Lymphocyte ratio, *SD* Standard deviation, *sDMARD* Synthehic Disease Modyfiying Anti-Rheumatic Drugs, *WBC* white blood cell (10^3^/mm^3^).

**Table 2:** Comparison of patients’ psychiatric scale scores according to treatments.

	sDMARD (n = 207)	sDMARD + Steroid (n = 285)	z value	p value
MDQ^a^	2 (0–13)	4 (0–13)	−5.7	**<0.001** *
HAD-D^a^	5 (0–19)	8 (0–19)	−6.1	**<0.001** *
HAD-A^a^	4 (0–17)	7 (0–18)	−5.4	**<0.001** *
mHCL^a^	8 (0–22)	10 (0–23)	−5.4	**<0.001** *

*sDMARD* Synthehic Disease Modyfiying Anti-Rheumatic Drugs, *HAD-A* Hospital Anxiety and Depression Scale-Anxiety, *HAD-D* Hospital Anxiety and Depression Scale-Depression; *mHCL* Modified Hypomania Checklist, *MDQ* Mood Disorder Questionnaire.

Continuous variables expressed as median (min-max) values.

a p value has been calculated using Mann Whitney U test.

* Significant at p < 0.05 level.

**Table 3:** Comparison of patients’ mood disorder scale scores of the patients according to the disease activity.

	Remission	Mild	Moderate and severe	p value
MDQ^a^	2.1 ± 2.8 / 1.6–2.6	1.72 ± 2.0 /1.3–2.1	5.4 ± 3.7 / 5.0–5.8	**<0.001** ^*^
HAD-D^a^	4.7 ± 3.5 / 4.1–5.3	4.5 ± 3.1 / 4.0–5.1	9.5 ± 4.6 / 9.0–10.0	**<0.001** ^*^
HAD-A^a^	5.3 ± 3.7 / 4.7–6.0	4.5 ± 2.7 / 4.0–5.0	8.2 ± 4.2 / 7.8–8.7	**<0.001** ^*^
mHCL^a^	7.2 ± 3.0 / 6.8–7.8	7.1 ± 2.6 / 6.6–7.6	11.3 ± 4.2 / 10.8–11.8	**<0.001** ^*^

*HAD-A* Hospital Anxiety and Depression Scale-Anxiety, *HAD-D* Hospital Anxiety and Depression Scale-Depression, *mHCL* Modified Hypomania Checklist, *MDQ* Mood Disorder Questionnaire, *SD* Standard deviation.

Unless otherwise states, values are presented as Mean ± SD / 95% CI.

a p value has been calculated using Mann Whitney U test with Bonferroni correction, significant at p < 0.017 level.

**Table 4:** Linear Regression Analysis of Mood Scales by HAQ and DAS28-CRP.

Scale		Beta	t	%95 CI	p value
mHCL	HAQ	0.189	2.55	0.004 – 0.031	**0.011** *
	DAS28-CRP	0.249	3.91	0.036 – 0.110	**<0.001** *
HAD-A	HAQ	0.042	0.62	−0.008 – 0.016	0.530
	DAS28-CRP	0.009	0.16	−0.031 – 0.036	0.875
HAD-D	HAQ	0.086	1.17	−0.005 – 0.019	0.242
	DAS28-CRP	0.271	4.3	0.038 – 0.102	**<0.001** *
MDQ	HAQ	0.155	1.72	−0.009 – 0.041	0.085
	DAS28-CRP	0.151	2.0	−0.018 – 0.121	**0.045** *

*DAS28-CRP* Disease Activity Score28-C-Reactive protein, *HAD-A* Hospital Anxiety and Depression Scale-Anxiety, *HAD-D* Hospital Anxiety and Depression Scale-Depression, *HAQ* Health Assessment Questionnaire, *mHCL* Modified Hypomania Checklist, *MDQ* Mood Disorder Questionnaire.

* Significant at p < 0.05 level.

## Data Availability

The datasets used and/or analysed during the current study are available from the corresponding author on reasonable request.
